# Sanitation, Stress, and Life Stage: A Systematic Data Collection Study among Women in Odisha, India

**DOI:** 10.1371/journal.pone.0141883

**Published:** 2015-11-09

**Authors:** Kristyna R. S. Hulland, Rachel P. Chase, Bethany A. Caruso, Rojalin Swain, Bismita Biswal, Krushna Chandra Sahoo, Pinaki Panigrahi, Robert Dreibelbis

**Affiliations:** 1 Center for Global Health, University of Chicago, Chicago, Illinois, United States of America; 2 Johns Hopkins Bloomberg School of Public Health, Baltimore, Maryland, United States of America; 3 Department of Behavioral Science and Health Education, Center for Global Safe Water, Rollins School of Public Health, Emory University, Atlanta, Georgia, United States of America; 4 Asian Institute of Public Health, Bhubaneswar, India; 5 Epidemiology and Pediatrics and Center for Global Health and Development, College of Public Health, University of Nebraska Medical Center, Omaha, Nebraska, United States of America; 6 Center for Applied Social Research, University of Oklahoma, Norman, Oklahoma, United States of America; Technion - Israel Institute of Technology, ISRAEL

## Abstract

Emerging evidence demonstrates how inadequate access to water and sanitation is linked to psychosocial stress, especially among women, forcing them to navigate social and physical barriers during their daily sanitation routines. We examine sanitation-related psychosocial stress (SRPS) across women’s reproductive lives in three distinct geographic sites (urban slums, rural villages, and rural tribal villages) in Odisha, India. We explored daily sanitation practices of adolescent, newly married, pregnant, and established adult women (n = 60) and identified stressors encountered during sanitation. Responding to structured data collection methods, women ranked seven sanitation activities (defecation, urination, menstruation, bathing, post-defecation cleaning, carrying water, and changing clothes) based on stress (high to low) and level of freedom (associated with greatest freedom to having the most restrictions). Women then identified common stressors they encountered when practicing sanitation and sorted stressors in constrained piles based on frequency and severity of each issue. The constellation of factors influencing SRPS varies by life stage and location. Overall, sanitation behaviors that were most restricted (i.e., menstruation) were the most stressful. Women in different sites encountered different stressors, and the level of perceived severity varied based on site and life stage. Understanding the influence of place and life stage on SRPS provides a nuanced understanding of sanitation, and may help identify areas for intervention.

## Introduction

Despite efforts to improve access to basic resources, 768 million people rely on unimproved drinking-water for daily consumption, and an estimated 2.5 billion people lack access to improved sanitation facilities [1]. The link between access to these basic resources and psychosocial outcomes is an emerging area of importance in global health research. A study in Ethiopia found that water insecurity was significantly associated with psychosocial distress (*r* = 0.22, *p* < 0.001; one sided test) [2]. In Bolivia, Wutich and Ragsdale found that gender and the process of accessing water resources were significantly associated with emotional distress citing fear, worry, anger, and bother [3].

Though the literature focuses on water insecurity, sanitation access presents similar psychosocial risks, particularly for women and girls. In Kenya, Henley and colleagues studied hair cortisol concentrations as a biomarker for chronic stress, finding that concentrations were significantly higher in women who reported feeling unsafe while collecting water or accessing sanitation [4]. In a study of mental health in urban slums in Bangladesh, Gruebner, et al. found that elements of the built environment including access to a better toilet facility were significantly associated with high quality of life scores (WHO-5 scores) [5]. In addition to navigating the built and physical environment for sanitation activities, women face daily struggles with social status, access to resources, and social conflicts [6–8]. Time of day and privacy contribute to sanitation-related stress [9]. Moreover, women may have to cope with violence [10,11] or sexual assault and rape [12–14] while completing sanitation-related behaviors.

The present study seeks to add to the emerging body of research on the impact and determinants of sanitation-related psychosocial stress (SRPS). Data for this study are part of a larger mixed-methods study exploring women’s relationship with sanitation in low-income, infrastructure-restricted settings in Odisha, India. We build upon an initial Grounded Theory study that provided an empirically based, conceptual understanding for SRPS among women of reproductive age in Odisha [15]. Findings from this study suggest that sanitation encompasses a range of behaviors specific to the local cultural context, including: ritual anal cleansing, menstrual management practices, bathing, and changing clothes prior to reentering the house after defecation. Sanitation-related psychosocial stressors arise when women are unable to perform these behaviors free from worry, fear, or anxiety. According to the conceptual model proposed in the study, there are three categories of stressors, environmental, social, and sexual / gender-based violence stressors, whose intensity is modified by a woman’s life stage, living environment, or access to sanitation facilities.

The current study aims to examine and compare stress as it relates to the *specific* sanitation-related behaviors as well as explore the relative frequency and severity of individual stressors that contribute to SRPS among a sample of women in Odisha. Recognizing that these sanitation-related behaviors and stressors are contextually bound and dynamic in nature, this analysis explores the differential impact of common psychosocial stressors on women living in different geographic settings and occupying differing social roles within the household and community.

We selected systematic data collection methods–a broad family of interviewing techniques originally intended to examine tacit knowledge in ethnography and cognitive anthropology–for use in this study [16]. These methods have been used to explore the boundaries and dimensions of specific cognitive domains that may be culturally defined or difficult to articulate, such as kinship terms [17] or medicinal classifications [18,19], and the internal systems of classification that individuals employ. Unlike open-ended interviewing or participant observations, systematic methods entail asking all respondents the same questions and analyzing responses according to emic categorization rather than those imposed by the researcher.

For the purposes of this study, the successive application of multiple systematic data collection methods allowed us to simultaneously examine the dynamic nature of sanitation-related behaviors, the relative degree to which these behaviors have contributed to psychosocial stress, and the frequency and severity with which women in the sample and women like them in the broader population have dealt with psychosocial stressors.

## Methods

### Study Sites

Access to sanitation in much of India remains scarce, and an estimated 44% of the population practices open defecation [1]. However, access to water and sanitation facilities may vary considerably by geographic context. Therefore, we chose three resource-poor geographic locations in Odisha to reflect differing access to sanitation infrastructure as well as differing social and cultural practices: urban slums, rural villages, and rural tribal villages with a large proportion of ethnically distinct residents. In the urban site, we interviewed women in two slums in Bhubaneswar, the capital of Odisha (population density of 2,134 people per square kilometer). Some slum residents had access to either privately owned or public latrines, but several participants still reported practicing open defecation. Rural women were selected from Khurda district, an agricultural region outside of Bhubaneswar (population density of approximately 800 people per square kilometer). Low-density, rural tribal villages were selected from Sundargarh District (population density of 216 people per square kilometer), where about half of the population belongs to scheduled tribes (*Adivasis*) recognized by the Indian government [20] including Oraron, Munda, and Kisan tribes. In local terms, “tribal” is used to describe both the geographically isolated regions and ethnic minority populations, and we use the term “tribal” when referring to women from this site. Both sanitation practices and access to infrastructure vary here compared to rural areas in Odisha, and tribal women were therefore expected to face unique sanitation challenges.

### Sample and selection of participants

We purposively sampled women from four life stages that are reflective of social and biological characteristics that influence a woman’s place in her household and community: 1) “Adolescents”: unmarried women aged 14–24 who had reached menarche and who lived with their parents and extended families; 2) “Newly married women”: married two years or less, the majority of whom had moved to a new social and physical geography to join the husband’s family household; 3) “Pregnant women”: women who identified as pregnant during data collection, for whom pregnancy changed their household roles and created distinct physical needs for sanitation; and 4) “Established adult women”: women between the ages of 25 and 45 who had been married more than two years, and were not currently pregnant. This sampling technique, while not providing a proportionally representative sample of the population of women in Odisha, offered us an opportunity to assess life stage-based variance in SRPS in a small sample.

### Data Collection

Volunteer community health workers affiliated with the Asian Institute for Public Health (AIPH) identified 20 women at each study site for participation in the study for a total of 60 participants. Our stratified, purposive sampling strategy ensured equal representation from each of the four life stage groups of interest (5 women per life-stage group per site) and a sample of latrine users and non-users similar to the general population. A team of four female interviewers trained in systematic data collection methods completed recruitment and data collection. Data were collected from April to May of 2014.

We carried out structured interviews that employed two systematic methods: pile sorting and ranking (S1 File). Pile sorting methods have traditionally been used to understand the internal organization of domains through the generation of graphical multidimensional scaling plots [21] or hierarchical clusters [19]. However, the flexibility of these methods to examine the categorization and organization of a range of topics has resulted in innovative adaptations to, for example, explore abstract concepts such as stress in children [22], perceptions of post-traumatic mental health [23], and gender roles [24]. Ranking and rating techniques have been used to develop measurement tools for wealth and wellbeing reflective of local understandings of economic security [25,26] and as participatory tools to engage residents in identifying and prioritizing needs in their communities [27].

Structured interviews began with basic demographic questions about the woman’s household, followed by a data collection module on sanitation *behaviors* and one on *stressors*. For *behaviors*, we identified a local taxonomy [28] of sanitation-related behaviors from our initial qualitative study [15] that included defecation, urination, menstruation, post-defecation cleaning (*dhua dhoi*), post-defecation bathing, changing clothes, and carrying water for use in sanitation. Field staff verbally presented participants with seven index cards each labeled with one of these specific-sanitation related behaviors, and explained each card to the respondent As interviewers introduced each card, women indicated if the behaviors were part of their typical routines (e.g. pregnant women could choose to include or exclude menstruation, but the choice was up to the participant and we stipulated no rules as to what was applicable). If not applicable, the card associated with a behavior was set aside and excluded from further data collection in the interview. Next, interviewers asked women to ‘rank’ *stress* associated with each behavior—most stressful to least stressful using a quick-sort ranking method [16] in which respondents organize items along a specific continuum. The rank order of cards was read back to the participant and recorded by the interviewer. Next, the interviewer shuffled the cards and asked respondents to rank behaviors by *freedom*–from the behavior they had the most freedom to choose when and how to practice to the least freedom. Rank order was again recorded (S1 Table).

For *stressors*, we presented women with index cards labeled with specific sanitation-related stressors and challenges identified in previously conducted in-depth interviews [15]. Interviewers again verbally presented each card, and women identified cards with stressors that they considered applicable to their typical routines, excluding those that were not applicable from the remaining questions. Next, interviewers asked women to ‘sort’ the cards into three piles based on how *frequently* they encountered the problem: always, sometimes, or rarely. The groupings were recorded and the interviewer shuffled the cards for the next question. Finally, participants were asked to ‘sort’ cards based on perceived *severity*: high, medium, or low. After each exercise, interviewers reviewed the rankings or piles and asked participants to describe their reasoning with open-ended questions (S2 Table).

Interviewers took detailed notes of both the ranking and sorting outcomes as well as participant responses. Ranking and sorting results were entered into a database (S1 Database), and open-ended questions were digitally recorded, transcribed, translated, and de-identified.

### Data analysis

For sanitation behaviors, ranking data on *stress* and *freedom* were modeled using rank-ordered logistic regression by maximum likelihood, specifically with the rologit command in Stata 13.1 [29]. Rank-ordered logistic regression is used to estimate the probability that an item–in our case, a sanitation behavior–would be ranked by a respondent as first along the characteristic of interest. Rank-ordered logistic regression accepts incomplete rankings, making it amenable to data where participants can discard some items or, as in our case, exclude inapplicable items, as long as we assume that omitted items are ranked lower along the trait of interest than all items that were retained. Unlike conditional logit models that only account for how often an item was ranked first among a set, rank-ordered logistic regression takes into account all ranks assigned to an item. Therefore, two items with equal numbers of first place rankings can be differentiated in the rank-ordered model based on how many second, third, *etc*. rankings they received.

*Frequency* and *severity* data regarding stressors arising during sanitation practice were interpreted as Likert-type scale ratings. We found that reporting and comparing percentages of “high severity” and “always” responses was sufficient to illustrate variations of concerns across groups.

### Ethical approval

Prior to the interviews, all participants provided written consent. For girls under 18, interviewers collected written assent from the participant and written consent from her parent. Participants were informed of their rights to terminate the interview at any time and to skip any questions or topics that they did not wish to discuss. Names and other identifiers collected during the interview were redacted during the transcription process and the original audio files destroyed. Ethical approval for this study was provided by the Ethical Review Committee at AIPH (ERC Protocol No. 2013–03) and the Institutional Review Board at Emory University (Protocol 00069418).

## Results

### Participant Characteristics

Table 1 presents characteristics of the 60 study participants by geographic site. Women ranged in age from 14 to 45 years old. The majority of the participants (73%) and all women in rural areas identified as Hindu. Access to a private or public latrine was limited; the majority of our participants did not have access to latrine facilities (63%) and were forced to practice open defecation. Latrine access was highest among participants in the urban population.

**Table 1:** Participant Characteristics.

	Rural	Urban	Tribal	All Sites
**Mean age in years (SD)**				
Adolescents	20.2 (2.3)	17.4 (2.2)	19.2 (0.5)	18.9 (2.1)
Newly married	23.6 (1.8)	20.4 (3.2)	21.6 (1.8)	21.9 (2.6)
Pregnant	23.4 (0.9)	22.4 (3.0)	23.8 (1.3)	23.2 (1.9)
Established adults	37.0 (7.7)	37.2 (2.3)	41.0 (5.5)	38.4 (5.5)
**Education completed, n (%)**				
None	-	4 (20%)	4 (20%)	8 (13%)
Some primary	2 (10%)	6 (30%)	3 (15%)	11 (18%)
Primary completed	5 (25%)	2 (10%)	5 (25%)	12 (20%)
Some secondary	7 (35%)	6 (30%)	2 (10%)	15 (25%)
Secondary completed	4 (20%)	-	3 (15%)	7 (12%)
Some tertiary/university	2 (10%)	2 (10%)	2 (10%)	6 (10%)
Tertiary/university completed	-	-	1 (5%)	1 (2%)
**Religion, n (%)**				
Hindu	20 (100%)	17 (85%)	13 (65%)	44 (73%)
Muslim	-	3 (15%)	-	3 (5%)
Christian	-	-	7 (35%)	13 (22%)
**Latrine Access, n (%)**				
Private latrine	8 (40%)	8 (40%)	2 (10%)	18 (30%)
Public latrine	-	4 (20%)	-	5 (7%)
Open defecation	12 (60%)	8 (40%)	18 (90%)	38 (63%)

### Sanitation behaviors

Table 2 presents the percentage of women in each geographic region who self-reported engaging in each of the seven sanitation-related behaviors of interest. We assessed whether or not women engaged in these activities to ensure that women only responded to issues that were pertinent to them in the subsequent exercises; these questions were not asked to compare habits of women in urban versus rural versus tribal areas. Women everywhere report defecation, urination, post-defecation cleaning (of the hands and feet), and bathing as part of normal sanitation practice. Women in rural areas reported less carrying water for sanitation purposes, since many use sites at or near open water sources or were more likely to walk to a pond or a river to complete their washing. Only 25% of women (all Hindu) in the tribal site reported changing clothes after defecation, a practice that women reported in previous qualitative interviews to be strongly linked to Hindu beliefs about ritual cleanliness [15].

**Table 2:** Percentage of women who reported engaging in activities overall and by geographic area.

	Overall	Rural	Urban	Tribal
**Defecation**	100%	100%	100%	100%
**Urination**	100%	100%	100%	100%
**Menstruation**	93%	95%	100%	85%
**Post-defecation Cleaning**	100%	100%	100%	100%
**Carrying Water**	87%	70%	90%	100%
**Bathing**	100%	100%	100%	100%
**Changing Clothes**	70%	95%	90%	25%

#### Stress

Table 3 shows results of the rank-ordered logistic regression analysis for stress and freedom, indicating the probability of a specific behavior being ranked first (most stressful, greatest freedom). We present the data in as raw a format as possible to encourage a more nuanced understanding of the responses than statistics such as modes and or measures of dispersion would supply.

**Table 3:** Probability that each activity would be ranked as having the most stress or freedom associated with it according to rank ordered logistic regression. Probabilities are presented overall and by geographic area, and by life course group. Stars are used to represent where p-values fell when comparing each item’s probability of being ranked first when compared to a reference item (marked as “(ref)”).)”.

		**Geographic Area**			**Life Course Group**			
	**Overall**	**Urban**	**Rural**	**Tribal**	**Adolescent**	**Newly Married**	**Pregnant**	**Est. Adult**
**STRESS**								
**Menstruation**	22%***	17%**	25%**	24%**	17%*	32%***	41%***	10%
**Defecation**	21%***	16%**	17%	31%***	30%**	11%	15%**	30%*
**Carrying Water**	19%***	33%***	15%	14%	14%	21%**	20%**	19%
**Bathing**	14%**	11%*	14%	14%	15%	13%*	9%	15%
**Post-defecation Cleaning**	11%	10%	11%	9%	15%	9%	8%	9%
**Urination**	7% (ref)	5% (ref)	9% (ref)	8% (ref)	7% (ref)	5% (ref)	4% (ref)	11% (ref)
**Changing Clothes**	5%	8%	10%	1%***	3%	9%	3%	5%
**FREEDOM**								
**Urination**	25%***	26%***	19%**	28%*	26%**	24%*	27%*	20%**
**Bathing**	19%***	22%***	17%**	15%	21%**	15%	17%	23%***
**Post-defecation Cleaning**	16%***	15%*	19%**	14%	17%*	12%	21%	15%**
**Defecation**	13%**	9%	12%	19%	14%	16%	8%	16%**
**Carrying Water**	11%	13%*	8%	12%	11%	15%	10%	9%
**Changing Clothes**	8%	9%	20%**	2% **	4%	9%	9%	13%
**Menstruation**	7% (ref)	6% (ref)	6% (ref)	10% (ref)	6% (ref)	10% (ref)	9% (ref)	4% (ref)
		**Geographic Area**			**Life Course Group**			
	**Overall**	**Urban**	**Rural**	**Tribal**	**Adolescent**	**Newly Married**	**Pregnant**	**Est. Adult**
**STRESS**								
**Menstruation**	22%***	17%**	25%**	24%**	17%*	32%***	41%***	10%
**Defecation**	21%***	16%**	17%	31%***	30%**	11%	15%**	30%*
**Carrying Water**	19%***	33%***	15%	14%	14%	21%**	20%**	19%
**Bathing**	14%**	11%*	14%	14%	15%	13%*	9%	15%
**Post-defecation Cleaning**	11%	10%	11%	9%	15%	9%	8%	9%
**Urination**	7% (ref)	5% (ref)	9% (ref)	8% (ref)	7% (ref)	5% (ref)	4% (ref)	11% (ref)
**Changing Clothes**	5%	8%	10%	1%***	3%	9%	3%	5%
**FREEDOM**								
**Urination**	25%***	26%***	19%**	28%*	26%**	24%*	27%*	20%**
**Bathing**	19%***	22%***	17%**	15%	21%**	15%	17%	23%***
**Post-defecation Cleaning**	16%***	15%*	19%**	14%	17%*	12%	21%	15%**
**Defecation**	13%**	9%	12%	19%	14%	16%	8%	16%**
**Carrying Water**	11%	13%*	8%	12%	11%	15%	10%	9%
**Changing Clothes**	8%	9%	20%**	2% **	4%	9%	9%	13%
**Menstruation**	7% (ref)	6% (ref)	6% (ref)	10% (ref)	6% (ref)	10% (ref)	9% (ref)	4% (ref)

No stars indicates p≥0.05

one star (*) indicates p<0.05

two stars (**) indicates p<0.01

three stars (***) indicates p<0.001.

Menstruation was most likely to be ranked as the most stressful behavior in our total population, followed by defecation and urination. However, the ranking of stress associated with these behaviors varied considerably according to geographic site. For example, menstruation was highly likely to be ranked as most stressful among rural and tribal women, but carrying water was the most stressful aspect of sanitation practice in urban areas. Tribal women were about twice as likely to rank defecation as most stressful compared to urban and rural respondents.

Stress rankings also varied by life stage. For adolescents, defecation was ranked as the highest stress, followed by menstruation, bathing, and post defecation cleaning. Menstruation was most likely to be ranked as high stress among newly married and pregnant women. Carrying water was also among the most stressful activities among newly married women, pregnant women and established adults.

#### Freedom

Daily sanitation activities take women out of the domestic environment in order to access latrines, fields for open defecation, or communal water sources. Women face restrictions dictating when and how they may practice these activities, such as when they leave the household, where they go, and whom they are allowed to go with. Table 3 presents the probability that a woman ranks a sanitation-related activity as the one she can practice with the most freedom. Overall, women had a high (25%) probability of ranking urination as the behavior with the most freedom, a pattern consistent among all of our geographic and life course groups. The two activities least likely to be ranked as having a high degree of freedom were changing clothes and menstruation.

We note some variation in freedom by geographic site and life stage group. Among rural women, the activity most likely to be ranked as having the highest degree of freedom was changing clothes, followed by urination, post-defecation cleaning, and bathing. When comparing across life stages, though urination is most likely to be ranked as most free by adolescents, newly married and pregnant women, established adults had a higher probability of ranking bathing as most free. Defecation was ranked with a relatively high degree of freedom for adolescents, newly married, and established adult women; however, this is the least likely to be ranked as most free among pregnant women, indicating that pregnant women may face greater restrictions associated with this practice based on their physical needs and the social and cultural restrictions accompanying pregnancy.

**Fig 1:**
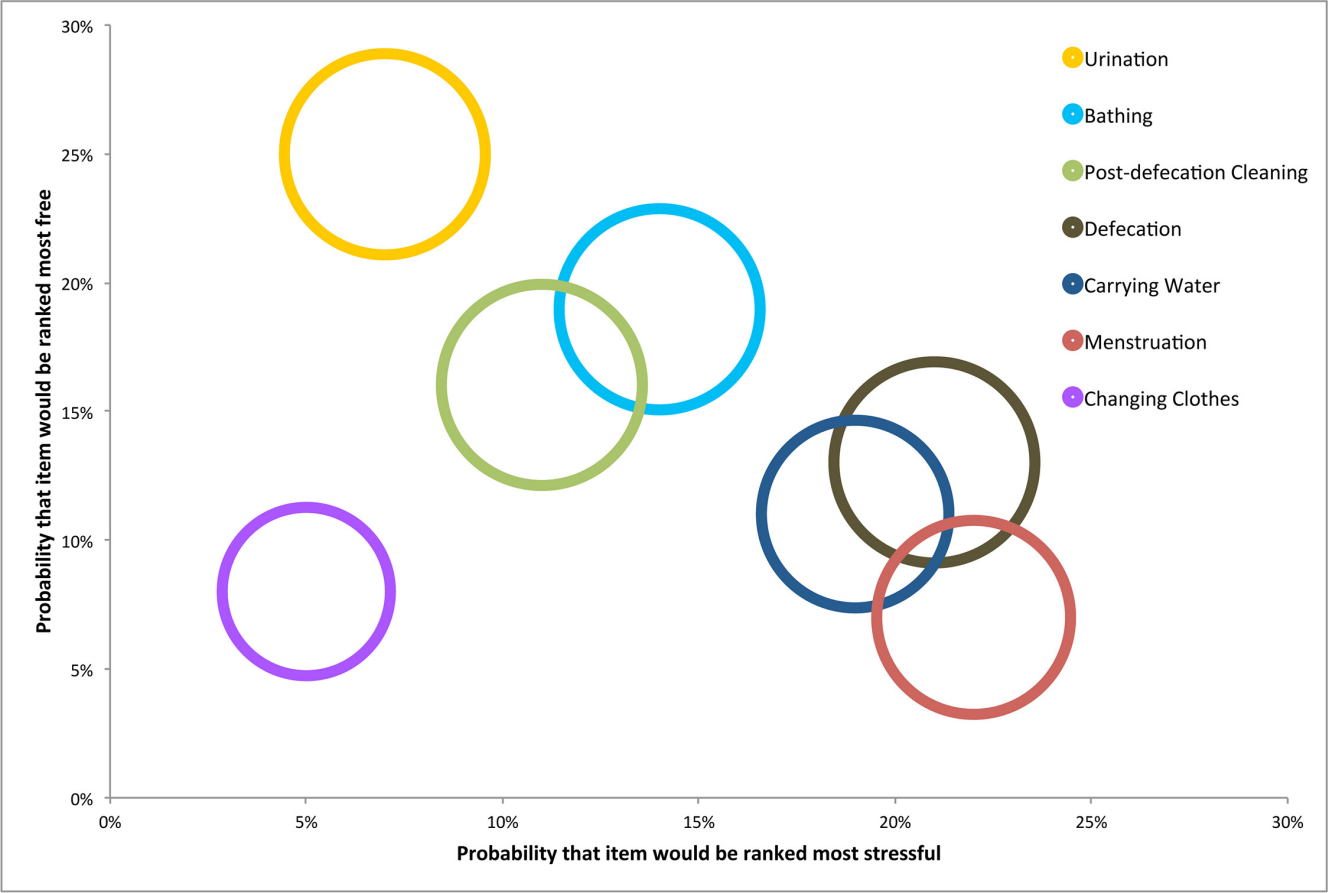
Overall applicability and ranking of stress and freedom associated with sanitation activities.

**Fig 2:**
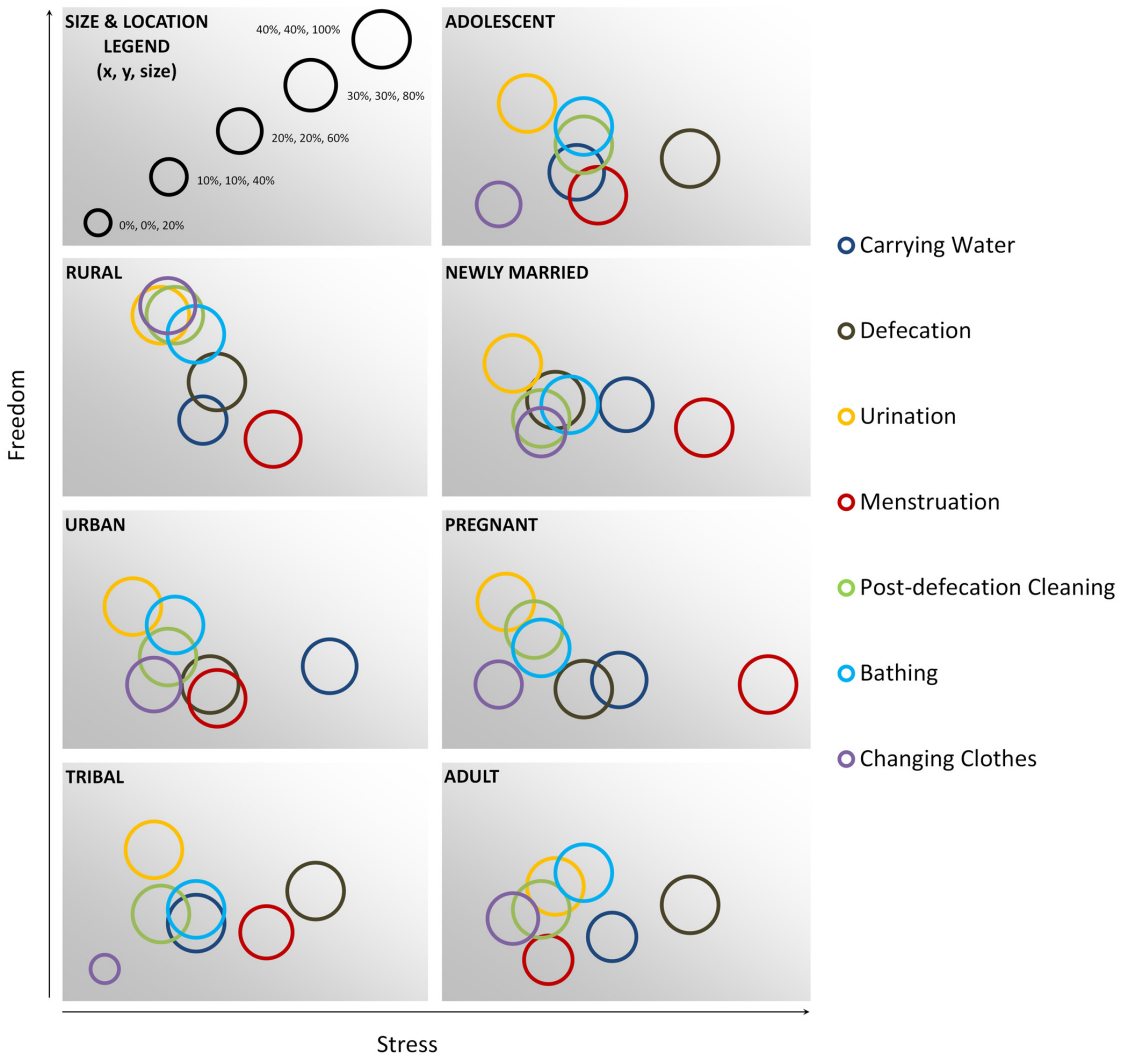
Applicability, stress, and freedom associated with sanitation activities. The diameter of each circle is proportional to the percentage of women who indicated the activity was applicable to them; the location of the center of the circle relative to the horizontal and vertical axes indicates the probability that the activity was rated most stressful and most free, respectively.

Fig 1 provides a visual representation of results, combining data on the percentage of women who reported completing specific behaviors (size of the circle), probability of a behavior being ranked as most stressful (x-axis), and the probability of a behavior being ranked as having the most freedom (y-axis). Fig 2 depicts this same visualization by life stage and geographic region. Among the total population, we note a clear and expected negative correlation between the probability that a behavior would be ranked as most stressful and as having the most freedom. Only changing clothes is an outlier from this general trend. This trend is less pronounced when visualizations are developed for each geographic area and for each life stage group. In particular, the graph of rural responses shows a steep association linking high freedom activities (such as urination and changing clothes) with lower stress compared to more restricted activities like menstruation with a high degree of stress. In the tribal site, the relationship between stress and freedom was less clear. However, the relative association between activities does follow the general trend (e.g. urination is higher in freedom and lower in stress than defecation, urination, and carrying water). Conversely, among adult women, the relationship between stress and freedom is slightly positive, and activities less likely to be associated with freedom are more likely to be associated with greater stress.

### Sanitation Stressors

We asked women to indicate what stressors they faced during sanitation (in general) based on twenty previously identified problems that were highly salient to women in Odisha [15]. Overall, women most commonly indicated rain (e.g., getting wet, walking through mud during sanitation), night/darkness, animals, and health during illness as sanitation stressors, with 87% or more of women indicating these were problems they faced. In all sites, women identified an average of 13 out of the 20 potential stressors as applicable to their sanitation practice. Table 4 summarizes the results of constrained pile sorting of stressors by frequency with which it is encountered (always, sometimes, rarely) and severity of the stressor (perceived severity of the stressor is high, medium, low).

**Table 4:** Probability that each stressor be ranked occurring most frequently or being ranked as high severity. Bolded values indicate stressors that have 50% or greater consequence in the category.

	**Overall Results**						**Rural**					**Urban**					**Tribal**		
**Stressor**	**Stressor is applicable**		**Stressor is always a concern**			**Stressor has high severity**	**Stressor is applicable**		**Stressor is always a concern**	**Stressor has high severity**		**Stressor is applicable**	**Stressor is always a concern**	**Stressor has high severity**	**Stressor is applicable**		**Stressor is always a concern**		**Stressor has high severity**
	*n*	*% of all respondents*	*% among "applicable" respondents*				*n*	*% of rural respondents*	*% among "applicable" respondents*			*n*	*% of urban respondents*	*% among "applicable" respondents*	*n*	*% of tribal respondents*	*% among "applicable" respondents*		
Rain	57	**95%**	12%	**58%**			18	**90%**	17%	**50%**	20	**100%**	10%	35%	19	**95%**	11%		**53%**
Night/dark	53	**88%**	32%	**72%**			18	**90%**	28%	**67%**	17	**85%**	**53%**	**76%**	18	**90%**	17%		**72%**
Encountering animals	52	**87%**	38%	**63%**			18	**90%**	44%	**56%**	15	**75%**	**60%**	**60%**	19	**95%**	16%		**74%**
Health (during illness)	52	**87%**	12%	**63%**			16	**80%**	31%	**56%**	17	**85%**	6%	**71%**	19	**95%**	0%		**63%**
Encountering ghosts	47	**78%**	**51%**	**68%**			16	**80%**	**56%**	**63%**	14	**70%**	**64%**	**64%**	17	**85%**	35%		**81%**
Post-natal	46	**77%**	17%	**59%**			13	**65%**	31%	**62%**	13	**65%**	15%	**69%**	20	**100%**	10%		47%
Being seen	45	**75%**	31%	32%			15	**75%**	33%	27%	14	**70%**	**57%**	**50%**	16	**80%**	6%		20%
Reputation	44	**73%**	**52%**	**68%**			16	**80%**	**56%**	**56%**	14	**70%**	**64%**	**86%**	14	**70%**	36%		**64%**
Encountering drunk people	43	**72%**	47%	**67%**			13	**65%**	38%	**62%**	16	**80%**	**69%**	**88%**	14	**70%**	29%		**50%**
Health (contracting infections)	41	**68%**	46%	**61%**			14	**70%**	43%	**64%**	16	**80%**	**56%**	**75%**	11	**55%**	36%		36%
Lack of safety	38	**63%**	46%	47%			9	45%	**56%**	44%	16	**80%**	**67%**	**56%**	13	**65%**	15%		38%
Rape/assault	36	**60%**	**58%**	**78%**			11	**55%**	36%	**64%**	14	**70%**	**86%**	**100%**	11	**55%**	45%		**64%**
Privacy	35	**58%**	40%	40%			12	**60%**	42%	42%	14	**70%**	36%	43%	9	45%	44%		33%
Distance	34	**57%**	**59%**	**59%**			9	45%	**67%**	33%	10	**50%**	40%	**70%**	15	**75%**	**67%**		**67%**
Restrictions going out	33	**55%**	37%	48%			10	**50%**	**60%**	40%	11	**55%**	45%	**64%**	12	**60%**	8%		42%
Males peeping	31	**52%**	23%	**61%**			10	**50%**	30%	40%	12	**60%**	25%	**75%**	9	45%	11%		**67%**
Receiving a scolding	27	45%	30%	48%			6	30%	17%	**50%**	13	**65%**	38%	7%	8	40%	25%		38%
Males teasing/throwing stones	23	38%	9%	35%			7	35%	0%	0%	9	45%	22%	44%	7	35%	0%		**57%**
Lack of space	21	35%	**90%**	**75%**			5	25%	**100%**	**75%**	13	**65%**	**92%**	**69%**	3	15%	**67%**		**100%**
Physical barriers	8	13%	38%	38%			1	5%	0%	0%	6	30%	**50%**	**50%**	1	5%	0%		0%
	**Adolescents**						**Newly Married**					**Pregnant**				**Established Adults**			
**Stressor**	**Stressor is applicable**		**Stressor is always a concern**		**Stressor has high severity**		**Stressor is applicable**		**Stressor is always a concern**	**Stressor has high severity**		**Stressor is applicable**	**Stressor is always a concern**	**Stressor has high severity**	**Stressor is applicable**	**Stressor is always a concern**	**Stressor has high severity**		
	*n*	*% of rural respondents*	*% among "applicable" respondents*				*n*	*% of urban respondents*	*% among "applicable" respondents*			*n*	*% of tribal respondents*	*% among "applicable" respondents*	*n*	*% of tribal respondents*	*% among "applicable" respondents*		
Rain	15	**100%**	13%		33%		12	**80%**	8%	**75%**		15	**100%**	7%	**80%**	15	**100%**	20%	47%
Night/dark	13	**87%**	46%		**69%**		11	**73%**	9%	**73%**		15	**100%**	47%	**67%**	14	**93%**	21%	**79%**
Encountering animals	13	**87%**	23%		**58%**		12	**80%**	**50%**	**50%**		14	**93%**	**50%**	**79%**	13	**87%**	31%	**69%**
Health (during illness)	14	**93%**	14%		**64%**		12	**80%**	8%	**67%**		13	**87%**	8%	**69%**	13	**87%**	15%	**54%**
Encountering ghosts	11	**73%**	27%		**63%**		12	**80%**	**58%**	**55%**		14	**93%**	**71%**	**86%**	10	**67%**	40%	**70%**
Post-natal	10	**67%**	40%		40%		11	**73%**	9%	**50%**		14	**93%**	21%	**79%**	11	**73%**	0%	**55%**
Being seen	12	**80%**	25%		25%		10	**67%**	30%	44%		12	**80%**	33%	33%	11	**73%**	36%	27%
Reputation	12	**80%**	**67%**		**83%**		12	**80%**	**50%**	**83%**		10	**67%**	**50%**	40%	10	**67%**	40%	**60%**
Encountering drunk people	13	**87%**	31%		**77%**		9	**60%**	**56%**	44%		11	**73%**	45%	**55%**	10	**67%**	**60%**	**90%**
Health (contracting infections)	12	**80%**	**50%**		**67%**		10	**67%**	**70%**	**50%**		12	**80%**	**50%**	**67%**	7	47%	14%	**57%**
Lack of safety	10	**67%**	40%		40%		8	**53%**	**50%**	38%		11	**73%**	45%	**64%**	9	**60%**	**50%**	44%
Rape/assault	12	**80%**	**67%**		**92%**		8	**53%**	**63%**	**63%**		9	**60%**	**56%**	**78%**	7	47%	43%	**71%**
Privacy	9	**60%**	**56%**		**55%**		7	47%	29%	**57%**		9	**60%**	44%	33%	10	**67%**	30%	20%
Distance	6	40%	**50%**		33%		10	**67%**	40%	40%		9	**60%**	**78%**	**89%**	9	**60%**	**67%**	**67%**
Restrictions going out	10	**67%**	**60%**		**50%**		9	**60%**	22%	**56%**		8	**53%**	38%	38%	6	40%	17%	**50%**
Males peeping	8	**53%**	13%		**50%**		8	**53%**	25%	**63%**		7	47%	29%	**57%**	8	**53%**	25%	**75%**
Receiving a scolding	8	**53%**	25%		25%		7	47%	43%	**71%**		6	40%	17%	33%	6	40%	33%	**67%**
Males teasing/throwing stones	9	**60%**	11%		**56%**		5	33%	20%	20%		3	20%	0%	0%	6	40%	0%	33%
Lack of space	6	40%	**100%**		**80%**		5	33%	**50%**	**60%**		5	33%	**100%**	**60%**	5	33%	**100%**	**100%**
Physical barriers	1	7%	**100%**		0%		2	13%	**50%**	**50%**		2	13%	0%	50%	3	20%	33%	33%

#### Frequency and Severity

Overall, the issues more likely than not to be considered applicable, as “always” a concern, and as stressors of high severity were rape/assault, distance, reputation, and ghosts. For the minority who considered it applicable, lack of space was also predominantly considered a persistent and severe concern. These stressors span multiple domains related to sanitation-related psychosocial stress [15] including the built and social environments. Lack of space and distance stand out as especially prominent sanitation infrastructure-related concerns, compared to physical barriers. Rape/sexual assault and reputation are distinguished from, for example, being scolded as particularly poignant constructs of the social environment that induce SRPS. Among the most concerning of stressors, we also find an example from the domain of cultural beliefs, namely, encountering ghosts.

The types of stressors and the frequency and severity with which they were encountered ranged by geographic site and life stage group (Table 4). While the majority of women in all sites and life stage groups reported the majority of the 20 stressors as applicable (ranging from 13 among established adult women to 17 among adolescents, and from 14 among tribal and rural women to 18 among urban women), the variation in describing those stressors as frequent or severe manifests the importance of understanding the context in which women encounter SRPS. For example, urban women identified physical barriers (like fences or gates restricting access to sanitation) as more applicable to their sanitation behaviors than rural or tribal women (30% in urban sites as opposed to 5% in both rural and tribal sites), and half of urban women rated physical barriers as a high severity concern (compared to 0% of rural and tribal women). Rape and sexual assault was particularly salient in the urban group where 70% of women said it was a stressor. Among these urban women, 86% were always concerned about it and 100% described it as a highly severe issue. In comparison, only 55% of rural and tribal women identified rape/sexual assault as applicable, and among these women it was not categorized as “always a concern” (36% of rural and 45% of tribal women) and 64% of women in both groups said it was highly severe. Being seen, a construct of the social environment, had roughly equal applicability across groups (14–16 of 20 women in each site marking it as applicable), but happened infrequently among women in the tribal site (only 6% said it was always a concern in tribal areas, compared to 57% in urban areas and 33% in rural areas) and seldom considered severe (20% of tribal women said it was a severe concern, compared to 50% of urban and 27% of rural women who considered it applicable). Males teasing or throwing stones was also similarly applicable across geographic sites (7–9 women per site), but varied greatly from rural women indicating that, even when applicable, it was never a high severity stressor nor one that was always a problem (0% of rural women categorized this in the most severe or frequent categories); tribal women agreed that males teasing or throwing stones was not always a problem, but, when it was, it was severe (57% indicated it was high severity).

The salience of specific stressors also changed by life stage. Rape was salient to a majority of women in all groups, but in no group was it as often considered salient, frequent, and severe as it was among adolescents. Reputation was a concern shared almost equally by adolescents and newly married women, with 80% in both groups considering it salient and 83% in both groups considering it high severity; half of newly married women and 67% of adolescents viewed it as always a concern. Pregnant women were especially concerned with issues that they perceived to be detrimental to their pregnancies, such as encountering ghosts–a concern that was not as often salient, frequent, or high severity in other life course groups.

**Fig 3:**
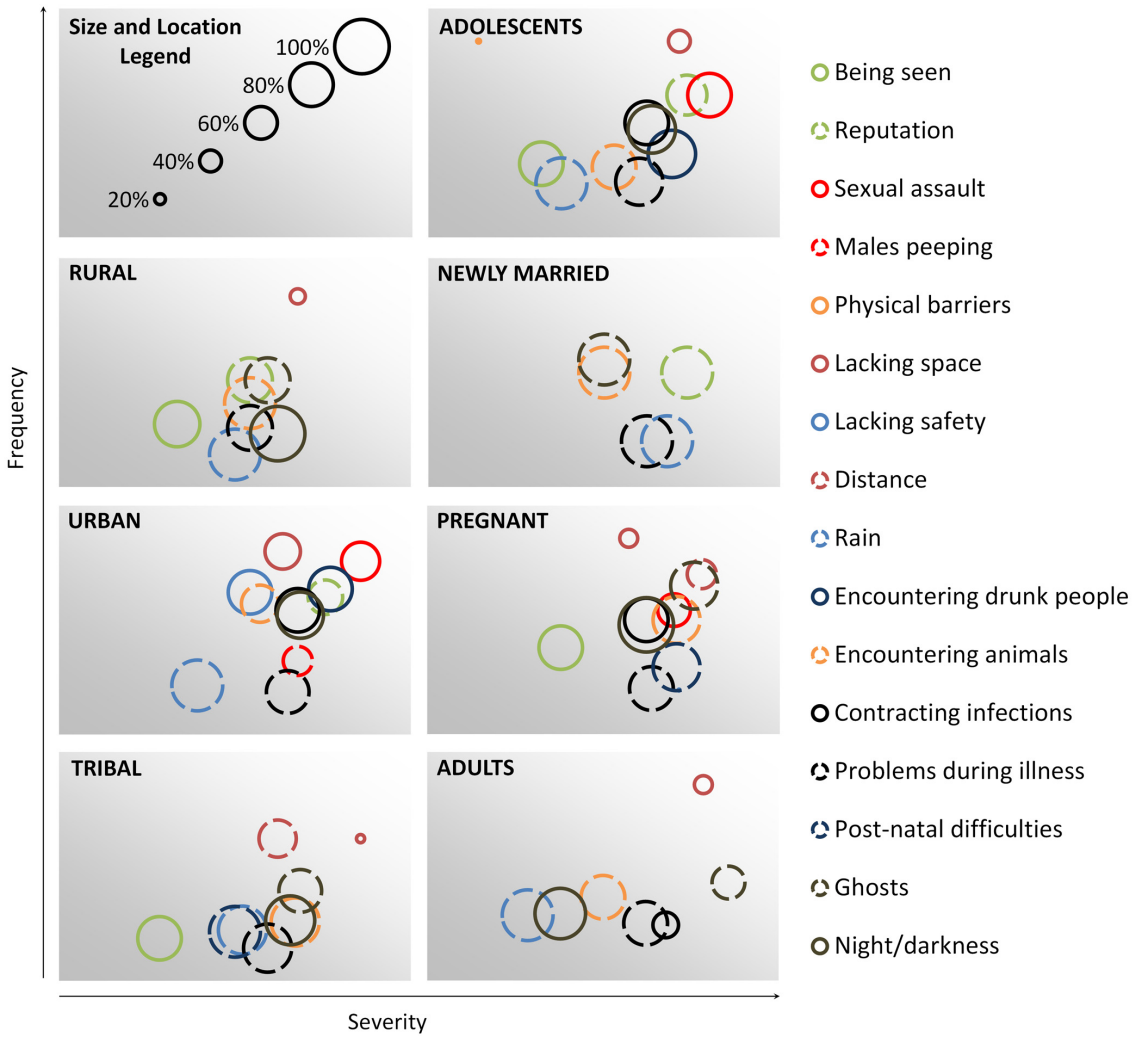
Visualizing Frequency and Severity of Common Stressors Based on Life Stage and Geographic Site. Each circle represents a sanitation stressor. The diameter of the circle is proportional to the percentage of women who reported that the stressor was applicable to them. The location of the midpoint of the circle on the horizontal and vertical axes reflects the proportion of those women who indicated that the item was a high severity stressor and high frequency stressor, respectively. Only stressors that were highly applicable, severe, or frequent are included in each graph.

We note a general positive trend between the perceived severity and perceived frequencies of stressor: issues that were commonly ranked as highly severe were also commonly ranked as issues they “always” encounter (Fig 3). Lack of space (in all geographic sites), sexual assault (among urban women), and distance (among tribal women) were likely to be ranked as both high frequency and high severity issues. Visualizing results also shows us exceptions to this relationship. For example, when applicable, adolescents encountering physical barriers ranked them as something they always encounter, however, this was not likely to be ranked as a severe stressor. Likewise, adult women who ranked “ghosts” as a stressor, were not likely to rank them as a frequent stressor, but they were often ranked as a high severity issue.

## Discussion

Using structured data collection methods for this research allowed us to explore the scope and dimensions of key sanitation-related stressors in a more nuanced manner than a survey would afford and more systematic than exploratory qualitative research. Ranking sanitation-related behaviors from most stressful to least stressful helped us to explore how stress manifests across sanitation activities. Women consistently ranked menstruation and carrying water as highly stressful activities, contributing to SRPS. Water is an essential component of sanitation related behaviors in this setting and was used in post-defecation cleaning, bathing and for menstrual hygiene management [15]. In urban areas, women usually rely on shared, public water sources that may be intermittently available, and the burden of collecting and carrying water to a site for defecation or urination was highly problematic. Despite the links between carrying water and other sanitation behaviors, water and sanitation provision in India are often operationalized independently. The delivery and provisioning of water may be coordinated by a state’s Department of Public Health and Engineering or by the State Water Board; however, different state-level departments may implement sanitation programs. In theory, India’s Total Sanitation Campaign (TSC, 1999–2012) aimed to incentivize user- and community-driven demand for sanitation, but the focus on infrastructure development has been criticized as a top-down, government-led approach [30]. The Swachh Bharat Mission (SBM), the recently launched government-led sanitation campaign in India, has committed billions of dollars to improve sanitation coverage through infrastructure development, user incentives, and community-mobilization. However, efforts remain targeted on sanitation infrastructure at the household-level. Though the nonprofit and private sectors play a role in increasing water, sanitation and hygiene services throughout the country, our data show that sanitation behaviors rely heavily on water access, suggesting the need for coordinated interventions among different levels of government and the public and private sector that respond to the social and physical needs of the users.

Furthermore, the majority of sanitation interventions focus on defecation and fecal management and often ignore other sanitation related behaviors like washing and menstrual hygiene. In addition, though the psychosocial implications of menstruation and menstrual management have been documented among adolescent girls [31–34], few studies have critically examined the psychological, interpersonal, and social repercussions among older populations. Our data highlight that stress related to menstrual management is particularly salient among newly married and pregnant women. Newly married women described that menstruation is highly stressful because they are new in their households and have to curtail their regular activities based on cultural traditions restricting sanitation behaviors, they feel uncomfortable talking about menstruation with their husbands and in-laws, and the physical symptoms associated with menstruation inhibit their normal activities. Similarly, pregnant women described menstruation as highly stressful, even though they were not currently experiencing monthly periods. Newly married and pregnant women living in their in-laws’ households face social restrictions surrounding menstruation and all sanitation-related behaviors such as restricted water access and taboos related to sexual intercourse, cooking, or religious practices during their periods [35,36]. Correspondingly, menstruation was also the least likely to be associated with a high degree of freedom among these women. Strategies that women may have had as adolescents may need to be renewed upon marriage and relocation into a new household.

Our results highlight the dimensionality of sanitation-related of stressors. We found that even stressors that occur less frequently may still be high severity issues, and that the intensity of stressors vary by life stage and geographic location. Examining stress and food security, a recent Food and Agriculture Organization (FAO) study found a relationship between severity and frequency, discussing how more severe indicators of food security (e.g. “Adult did not eat for a whole day”) are less frequently noted than less severe items (e.g. “Adult cut the size of meals) [37]. In our study, we similarly found that fewer women encountered some of the stressors that were most severe. For example, sexual assault was not commonly included as applicable, but when included, it was likely to be ranked as a high severity, high frequency issue, especially for adolescents and in urban areas. Violence that occurs due to inadequate access to water, sanitation, and hygiene facilities is of increasing concern in the water, sanitation, and hygiene community. Recently, rape and sexual assault associated with sanitation have received more attention in Indian media, explicitly linking lack of sanitation facilities with violence, rape, and lack of safety for women [38–40]. A review of literature examining gender-based violence and WASH shows how sensitivity, secrecy, and the complexity of violence inhibits the collection of reliable data, and the authors advocate for building an evidence base grounded in systematic, ethical evaluation of WASH related violence [41]. Our research identified violence and sexual assault as high severity stressors, but further research is needed to quantify the scope of the problem and suggest interventions.

Beyond the physical and social stressors associated with sanitation, this study illustrated how fear of ghosts was also perceived to be highly severe, especially among rural, pregnant and adult women. The high severity of this issue may be due local, traditional beliefs linking miscarriage to encounters with ghosts. Though we were unable to find studies specific to Odisha, an ethnographic study by Pauline Mahar Kolenda of sweepers in North India discusses a range of anxieties related to ghost and supernatural encounters, including the attribution of miscarriages to malevolent female ghosts [42]. This example highlights the usefulness of examining the stratification of stressors, especially when culturally significant proscriptions impact sanitation behaviors.

Understanding the dynamic sanitation behaviors, stressors, and the attributed level of severity is essential for informing practitioners about the context and implications of intervention. Identifying how stressors are related to location and life stage may help assign priorities in creating safe sanitation spaces. For example, for newly married women, physical barriers were less likely to be ranked as highly severe than for women in other life stage groups. Women in our study occasionally mentioned special places near the home where newly married women could defecate, and in some cases improvements to the home are used in negotiating a marriage. In rural Haryana, India, access to sanitation was used as bargaining power in a campaign called “No Toilet, No Bride,” minimizing social restrictions for newly married women during sanitation and improving standards for sanitation access [43]. This example suggests that interventions focused on physical barriers are more greatly needed for adolescent, pregnant, and established women than for newly married women.

In advocating for a contextualized, gender sensitive approach to sanitation, our research findings inform future study of SRPS, illustrating key differences across life stages and social settings. Additionally, given the numerous ways women experience stress related to sanitation, further study may illuminate factors that ameliorate stress. Using systematic data collection techniques helps to populate a range of factors and then explore them to identify relevance, key priorities, and more nuanced dimensions like stress, severity, and frequency. Women in different parts of India face a distinct constellation of stressors and their severity depending on physical surroundings, life stage, and access to sanitation facilities. Understanding the dynamics of how social geographies and life course stages shape women’s sanitation experience may help to tailor sanitation needs given cultural and geographic diversity.

## Strengths and Limitations

The systematic data collection methods employed in this study helped us to explore sanitation related psychosocial stress using an interactive format and generating comparisons between women of different ages living in different geographic locations. The results highlight some key areas that can help to inform future research on sanitation related to mental health; however, more research is needed to develop locally relevant psychometric scales. We recruited five women per life stage group per site for 60 total participants, allowing us to examine results in both social and geographic groupings. However, a larger sample size may afford more granularity in examining trends by life stage group and geographic site simultaneously (i.e. urban adolescents vs. tribal adolescents). It would also be valuable to explore the relationship between freedom and stress using a larger sample size. Additionally, some of the sanitation behaviors and stressors are shaped by cultural practices and socially defined roles, so the generalizability of some of our findings may be limited to low-resource settings of India.

## Conclusions

Factors contributing to SRPS differ by life stage and geographic site, and the context of sanitation must be understood to inform successful sanitation interventions. Understanding the network of factors, relationships and activities influencing mental health and feelings of distress gives us a more nuanced understanding of the ways women negotiate their sanitation environments. Further research measuring SRPS may help to significantly inform sanitation interventions, signposting key areas for infrastructural development and behavior change messaging.

## Supporting Information

S1 Database SDC sanitation stress database. This file provides data from all participant interviews and corresponds to questions in the interview guide.
(XLSX)

S1 File Interview Guide (in English). This guide was translated into Odiya for use during the interviews.
(DOCX)

S1 Table Operational definitions of sanitation-related behaviors. This table provides definitions of sanitation-related activities and behaviors described by participants.
(DOCX)

S2 Table Operational definitions of stressors. This table provides definitions of stressors encountered during sanitation as described by participants.
(DOCX)
