# Supplementary material for: Sanitation, Stress, and Life Stage: A Systematic Data Collection Study among Women in Odisha, India
Source: PLoS One. 2015 Nov 9;10(11):e0141883. doi: 10.1371/journal.pone.0141883 (PMC4638353; doi:10.1371/journal.pone.0141883)
Supplement: S1 File — This guide was translated into Odiya for use during the interviews. (DOCX) [file pone.0141883.s002.docx]

Systematic Data Collection Interview Guide

SHARE Aim 1 Sanitation Related Psychosocial Stress

Table of Contents

Section A. Participant Information & Consent 2

Section B. Sanitation-related activities 2

B1. Selection of cards 2

Ranking - Quick sort 2

Constrained pile sort 3

Section C: Challenges faced during defecation 3

C1. Selection of cards 3

Rating 4

Constrained pile sort 4

Data entry form 5

# Section A. Participant Information & Consent

Administration of consent as per interview guidelines.

Collection of participant information.

Introduction:

*The following introductions, questions and instructions have been translated into Odiya in consultation with the data collection team. The data collectors will employ setting appropriate and/or emic terms whenever possible.*

We have been talking with women in Odisha about sanitation related stress and problems women face. Today, we would like to ask you about your experiences with these issues. First, we’ll start by asking you if a set of items applies to your situation. When we ask you about these things, please answer honestly if you understand the term and if it relates to your experience with sanitation.

Then, we’ll ask you a number of questions with these items written on cards. We will ask you to the cards multiple times, but the questions will be different. Please answer to the best of your opinion. There are no wrong answers.

# Section B. Sanitation-related activities

First, we will begin by asking you about activities that relate to sanitation. Please tell us if this is something you do.

## B1. Selection of cards

*(Data collector presents cards one by one and participant is asked to say either “yes” or “no” if this activity does not apply to the participant).*

1. Carrying water
2. Defecation
3. Urination
4. Menstruation
5. Post-defecation cleaning
6. Bathing
7. Changing clothes

## Ranking - Quick sort

B2. STRESS: Given the sanitation activities that you say you do, we are interested to know which activities cause stress or tension in your life. Order activities from more stress to less stress.

*(This method will use a quick sort technique using the first activity as an index. Anything more stressful than the first card will go in a higher stress pile, and things less stressful will go in a less stress pile. The items in the high stress pile will be compared and ordered. Then the lower stress pile will be compared and ordered. Finally, the participant will review the order to ensure it was correctly sorted.)*

Follow-up questions: You say *n*  is high stress. Tell me why this causes you stress? Why does *x* cause you the least stress?

B3. FREEDOM: Women have told us that there are rules or restrictions surrounding some sanitation activities. Please order these sanitation activities from the ones that you feel you have the most freedom to do when and how you want to, to the activities where you feel there is the least freedom (or there are the most restrictions).

Follow-up questions: Why do you feel *x, y* give you the most freedom? How does this make you feel? You say that *p, q*  are activities with the most restrictions. Can you tell me about these restrictions? How do these make you feel?

# Section C: Challenges faced during defecation

In this section, we will be asking you about challenges you may face when going for defecation. These cards have challenges that other women have mentioned. Please indicate if these challenges apply to your situation or not.

## C1. Selection of cards

*(Data collector presents cards one by one and participant is asked to say either “yes” or “no” if this challenge does not apply to the participant).*

1. being seen by people
2. males (teasing, throwing things)
3. scolding
4. restrictions on when to go out
5. reputation
6. rape/sexual assault
7. males peeping / revealing
8. fences/ physical barriers
9. lack of space
10. privacy or isolation
11. lack of safety
12. distance
13. rainy– getting wet/muddy
14. drunk people
15. animals (cows, dogs, snakes, etc)
16. health / infections
17. health / during illness
18. post-natal problems
19. ghosts
20. night / darkness

## Rating

C2. SEVERITY: How severe of a problem is this issue for you during defecation? Place each card in a pile indicating:

Pile 1: High (This problem is very serious to me)

Pile 2: Medium (This is a serious problem, but not one of the most serious)

Pile 3: Low (This is a problem, but it is not serious)

Follow-up questions – Tell me about the cards you put in the “high severity” pile. Can you explain why these challenges are serious to you? Can you tell me about the cards in the “low” pile? Why do you think these challenges are less serious?

## Constrained pile sort

C3. FREQUENCY: How often do you feel you face these challenges? How often are these things you are concerned about when you go for defecation? Sort into the pile you think is the best description of how frequently you face these challenges.

Pile 1: Always (I worry about this problem every time I go for defecation)

Pile 2: Sometimes (depends on the time of day or the season, but this is not a challenge all the time)

Pile 3: Rarely (I face this challenge infrequently and it rarely comes up)

Follow-up questions – Describe your piles: How did you choose which to put in each pile? Compare the challenges in terms of severity and frequency?

# Data entry form

| **Part A. Participant information**  Consent form administered | | | | |
| --- | --- | --- | --- | --- |
| **A01.** | Total interview time: ­_____________ Time (start) __:__ Time: (end) __:__ | | | |
| **A02.** | Interview ID:  _________________________  (Example: SDADR010414KH) | **A03.** Consent Provided: ☐ 1. Yes ☐ 2. No | | |
| **A04.** | Participant Type / Life stage: ☐ 1. Adolescent, unmarried, living with parents ☐ 3. Pregnant  ☐ 2. Newly Married ☐ 4. Adult woman | | | |
| **A05.** | Study area: ☐ 1. Rural ☐ 2. Urban ☐ 2. Tribal | | | |
| **A06.** | Age: ___________ | | | |
| **A07.** | Education: ☐ 1. None ☐ 5. Secondary Completed  ☐ 2. Some Primary School ☐ 6. Some Tertiary / University  ☐ 3. Primary Completed ☐ 7. Tertiary / University Completed  ☐ 4. Some Secondary | | | |
| **A08.** | Number of years living in community: _____ | | **A09.** | Religion: _______________________ |
| **A10.** | Caste: ____________________________ | | **A11.** | Has BPL Card: ☐ 1. Yes ☐ 2. No |
| **A12.** | Latrine access ☐ 1. Yes (private) ☐ 2. Yes (public) ☐ 3. No  **🡪 A11_3. If No,** check all defecation places that apply  ☐ 1. Open field (Private) ☐ 6. Canal side  ☐ 2. Open field (Public) ☐ 7. Railway side  ☐ 3. Jungle ☐ 8. Road side  ☐ 4. Agricultural land ☐ 9. On polythene or paper for later disposal  ☐ 5. River side ☐ 10. Back of house | | | |
| **A13.** | Marital Status (check one):  ☐ 1. Unmarried  ☐ 2. Married **🡪 A12_1. If Married,** number of years married: ______________  **🡪 A12_2. If Married,** do you live with your husband  ☐ 1. Yes, live with husband  ☐ 2. No, husband works outside the home  ☐ 3. No, Divorced **🡪A12_3. If Divorced,** # of years divorced: ______  ☐ 4. No, Separated **🡪A12_4. If Separated,** # of years separated: ____  ☐ 5. No, Widowed **🡪A12_5. If Widowed,** # of years widowed: _____ | | | |
| **A14.** | Number of People in the HH: ___________ | | **A15.** | Is it a joint family? ☐ 1. Yes ☐ 2. No |
| **A16.** | Do you have any children? ☐ 1. Yes ☐ 2. No  **🡪 A15_1 If YES,** how many children _____ | | | |
|  |  | | | |
| **INTRODUCTION:** We have been talking with women in Odisha about sanitation related stress and problems women face. Today, we would like to ask you about your experiences with these issues. First, we’ll start by asking you if a set of items applies to your situation. When we ask you about these things, please answer honestly if you understand the term and if it relates to your experience with sanitation. Then, we’ll ask you a number of questions with these items written on cards. We will ask you to the cards multiple times, but the questions will be different. Please answer to the best of your opinion. There are no wrong answers. | | | | |

| **Part B. Sanitation Activities**  First, we will begin by asking you about activities that relate to sanitation. Please tell us if this is something you do. | |
| --- | --- |
| **B01.** | Yes / applies- (Present cards one by one and participant is asked to say either “yes” or “no” if this activity does not apply to the participant).  ☐ 1. Carrying water ☐ 5. Post-defecation cleaning  ☐ 2. Defecation ☐ 6. Bathing  ☐ 3. Urination ☐ 7. Changing clothes  ☐ 4. Menstruation |
| **B02.** | **Ranking – Quick Sort**  STRESS associated with sanitation activities - Given the sanitation activities that you say you do, we are interested to know which activities cause stress or tension in your life. Order activities from more stress to less stress.   \| **Stress w/ sanitation** \| \| \| --- \| --- \| \| **Rank** \| **Card #** \| \| **B02.1** \|  \| \| **B02.2** \|  \| \| **B02.3** \|  \| \| **B02.4** \|  \| \| **B02.5** \|  \| \| **B02.6** \|  \| \| **B02.7** \|  \| |
|  | Follow-up questions: You say *n*  is high stress. Tell me why this causes you stress? Why does *x* cause you the least stress?  (notes) |
| **B03.** | **Ranking – Quick Sort**  FREEDOM: Women have told us that there are rules or restrictions surrounding some sanitation activities. Please order these sanitation activities from the ones that you feel you have the most freedom to do when and how you want to, to the activities where you feel there is the least freedom (or there are the most restrictions).   \| **Freedom for sanitation** \| \| \| --- \| --- \| \| **Rank** \| **Card #** \| \| **B03.1** \|  \| \| **B03.2** \|  \| \| **B03.3** \|  \| \| **B03.4** \|  \| \| **B03.5** \|  \| \| **B03.6** \|  \| \| **B03.7** \|  \| |
|  | Follow-up questions: Why do you feel *x, y* give you the most freedom? How does this make you feel? You say that *p, q*  are activities with the most restrictions. Can you tell me about these restrictions? How do these make you feel?  (notes) |
|  |  |

| **Part C. Challenges related to defecation**  In this section, we will be asking you about challenges you may face when going for defecation. These cards have challenges that other women have mentioned. Please indicate if these challenges apply to your situation or not. | | |
| --- | --- | --- |
| **C01.** | Yes / applies- (Present cards one by one and participant is asked to say either “yes” or “no” if this activity does not apply to the participant). | |
|  | ☐ 1. Being seen by people  ☐ 2. Males [teasing, throwing stones]  ☐ 3. Scolding  ☐ 4. Restrictions on when to go out  ☐ 5. Reputation  ☐ 6. Rape / sexual assault  ☐ 7. Males peeping / revealing  ☐ 8. Fences / physical barriers  ☐ 9. Lack of space  ☐ 10. Privacy or isolation | ☐ 11. Lack of safety  ☐ 12. Distance  ☐ 13. Rain [getting wet, mud, feces]  ☐ 14. Drunk people  ☐ 15. Animals [cows, dogs, snake, insect]  ☐ 16. Health (contracting infections)  ☐ 17. Health (during illness)  ☐ 18. Post-natal problems  ☐ 19. Ghosts  ☐ 20. Night / darkness |
| **C02.** | **Rating**  SEVERITY: How severe of a problem is this issue for you during defecation? Place each card in a pile indicating:   \| Pile \| Card # \| \| --- \| --- \| \| **HIGH** \|  \| \| **MEDIUM** \|  \| \| **LOW** \|  \| | |
|  | Follow-up questions – Tell me about the cards you put in the “high severity” pile. Can you explain why these challenges are serious to you? Can you tell me about the cards in the “low” pile? Why do you think these challenges are less serious?  (notes) | |
| **C03.** | **Constrained Pile Sort**  FREQUENCY: How often do you feel you face these challenges? How often are these things you are concerned about when you go for defecation? Sort into the pile you think is the best description of how frequently you face these challenges.   \| Pile \| Card #s \| \| --- \| --- \| \| **ALWAYS**  I worry about this problem every time I go for defecation \|  \| \| **SOMETIMES**  Depends on the time of day or the season, but this is not a challenge all the time \|  \| \| **RARELY**  I face this challenge infrequently and it rarely comes up \|  \| | |
|  | Follow-up questions – Describe your piles: How did you choose which to put in each pile?  (notes) | |
