# Supplementary material for: Sanitation, Stress, and Life Stage: A Systematic Data Collection Study among Women in Odisha, India
Source: PLoS One. 2015 Nov 9;10(11):e0141883. doi: 10.1371/journal.pone.0141883 (PMC4638353; doi:10.1371/journal.pone.0141883)
Supplement: S1 Table — This table provides definitions of sanitation-related activities and behaviors described by participants. (DOCX) [file pone.0141883.s003.docx]

S1: Operational definitions of sanitation-related behaviors

| **Sanitation Behavior** | **Description** |
| --- | --- |
| 1. Defecation | The act of defecating |
| 1. Urination | The act of urinating |
| 1. Menstruation | Menstruation and associated menstrual hygiene management |
| 1. Post-defecation cleaning | Washing of the hands, feet and anus after defecating |
| 1. Bathing | Bathing of the entire body |
| 1. Changing clothes | Changing clothes worn for defecation before reentering the home |
| 1. Carrying water | Gathering and toting water used for sanitation related activities |
