# Supplementary material for: Sanitation, Stress, and Life Stage: A Systematic Data Collection Study among Women in Odisha, India
Source: PLoS One. 2015 Nov 9;10(11):e0141883. doi: 10.1371/journal.pone.0141883 (PMC4638353; doi:10.1371/journal.pone.0141883)
Supplement: S2 Table — This table provides definitions of stressors encountered during sanitation as described by participants. (DOCX) [file pone.0141883.s004.docx]

S2. Operational definitions of stressors encountered during sanitation

| **Challenges** | **Description** |
| --- | --- |
| 1. Being seen by people | Lack of privacy during the practice of sanitation |
| 1. Males (teasing, throwing things) | Comments, taunts, or pestering by males during sanitation |
| 1. Scolding | Reprimands due to location, timing or duration of behavior |
| 1. Restrictions on when to go out | Household-imposed rules regarding the time of day when women are allowed to practice sanitation |
| 1. Reputation | The beliefs about a woman’s integrity of conduct with males, usually in relation to marriageability |
| 1. Rape/sexual assault | Unwanted sexual conduct or attempts to coerce women into sexual acts |
| 1. Males peeping / revealing | Inappropriate, deliberate observation of women in the act of sanitation when private parts may be exposed |
| 1. Fences/ physical barriers | Barriers surrounding a defecation, urination, or bathing site that impede access |
| 1. Lack of space | Crowded or small environments that limit the privacy and cleanliness of sanitation |
| 1. Privacy or isolation | Perceived vulnerability due to little privacy or being away from the safety of others |
| 1. Lack of safety | General feelings of unease or lack of safety |
| 1. Distance | The amount of space between the household and locations of sanitation behaviors |
| 1. Rainy– getting wet/muddy | Refers to muddy or dirty environments accessing or at the site of sanitation behaviors |
| 1. Drunk people | Encounters with people who have been drinking alcohol and act irrationally |
| 1. Animals (cows, dogs, snakes, etc) | Encounters with wild or domesticated animals that cause fear or irritation when seeking sanitation sites |
| 1. Health / infections | Concerns about contracting illnesses due to contact with the sanitation environment |
| 1. Health / during illness | Difficulties in accessing sanitation when one is ill (i.e. while having dysentery, malaria, etc.) |
| 1. Post-natal problems | Problems women or those assisting women face after pregnancy including incontinence or complications due to pain, tears, or infection to the anus, vagina, or perineum |
| 1. Ghosts | Fears of encountering ghosts or spirits or the perceived consequences of such encounters |
| 1. Night/darkness | Fears of navigating the sanitation environment during the night time |
